# Evaluation Algorithm of Root Canal Shape Based on Steklov Spectrum Analysis

**DOI:** 10.1155/2019/4830914

**Published:** 2019-11-03

**Authors:** Dongqing Wu, Jian Gao, Xiaoli Hu, Zhengtao Xiao, Zhuwei Huang, Lanyu Zhang, Xin Chen, Yunbo He

**Affiliations:** ^1^State Key Laboratory of Precision Electronic Manufacturing Technology and Equipment, Guangdong University of Technology, Guangzhou, 510006, China; ^2^School of Computational Science, Zhongkai University of Agriculture and Engineering, Guangzhou, 510220, China; ^3^Department of Operative Dentistry and Endodontics, Guanghua School of Stomatology, Hospital of Stomatology, Guangdong Provincial Key Laboratory of Stomatology, Sun Yat-Sen University, Guangzhou, Guangdong, 510055, China; ^4^School of Electro-Mechanical Engineering, Guangdong Polytechnic of Industry and Commerce, Guangzhou, 510510, China

## Abstract

In recent years, we have seen more and more interest in the field of medical images and shape comparison motivated by the latest advances in microcomputed tomography (*μ*CT) acquisition, modelling, and visualization technologies. Usually, biologists need to evaluate the effect of different root canal preparation systems. Current root canal preparation evaluation methods are based on the volume difference, area difference, and transportation of two root canals before and after treatment. The purpose of root canal preparation is to minimize the volume difference and ensure the complete removal of the smear layer. Previous methods can reflect some general geometric differences, but they are not enough to evaluate the quality of root canal shape. To solve this problem, we proposed a novel root canal evaluation method based on spectrum and eigenfunctions of Steklov operators, which can be served as a better alternative to current methods in root canal preparation evaluation. Firstly, the ideal root canal model was simulated according to the root canal model before and after preparation. Secondly, the Steklov spectrum of the two models was calculated. Thirdly, based on the spectrum and the histogram of the Gaussian curvature on the surface, the weight of each eigenvalue was computed. Therefore, the Steklov spectrum distance (SSD), which measures shape difference between the root canals, was defined. Finally, the calculation method that quantifies the root canal preparation effect of root canals was obtained. Through experiments, our method manifested high robustness and accuracy compared with existing state-of-the-art approaches. It also demonstrates the significance of our algorithm's advantages on a variety of challenging root canals through result comparison with counterpart methods.

## 1. Introduction

In recent years, imaging technology represented by 3D digital geometry technology has widely been used in the medical field. The 3D model can display critical information such as the three-dimensional topological structure, geometric information, and anatomical structure of the lesion more intuitively. It lays a solid foundation for successive operations, such as simulating the operation, human-computer interaction, quantitative morphometry, and telemedicine [1]. Researchers have paid more and more attention to the issues of segmentation, registration, difference evaluation, and classification retrieval of 3D medical models. With the popularity of measurement tools represented by *μ*CT, the evaluation of anatomical structure before and after root canal preparation has become a hot topic in medical imaging [2–9].

**Figure 1:**
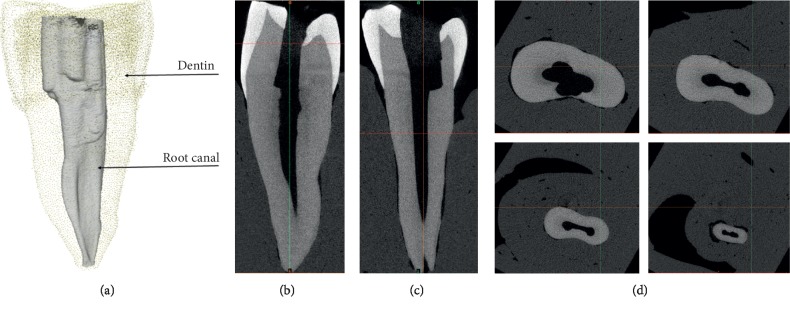
Root canal and dentin in the first premolar. (a) Root canal and dentin. (b) Left view. (c) Right view. (d) Four Dicoms for top to bottom.

The root canal is the part of the cavity in the middle of the tooth. The upper part of the cavity is broad, called the pulp cavity, and the lower part has a tubular structure from which the blood vessels of the dental nerve and the nutrient nerve are derived, as shown in Figure 1. In [10], Vertucci investigated 2,400 permanent teeth and categorized them into eight types. He studied the morphology and root canal of adult and established the evaluation criteria of treatment difficulty. From the point of view of dental anatomy, root canal cleansing and complete obturation with an inert filling material are very necessary when the root canal morphology of teeth is affected. Root canal therapy generally consists of three stages: root canal preparation, root canal disinfection, and root canal obturation. Root canal preparation is the critical step for root canal obturation therapy, including root canal cleaning and formation. Before root canal preparation, the location, length, shape, and number of root canal orifices need to be determined. Under the guidance of the dental operating microscope, ultrasonic root canal files and trumpet hand files are used to probe the root canal to the apical orifice. Root canals with small or blocked root canals should be cleaned and dredged with ethylenediaminetetraacetic acid (EDTA) or other solutions. Infected dentin should be removed and sterilized by root canal instruments to facilitate root canal obturation. The prepared root canals should contain the original root canal shape, retain the narrow part of the apex, and form the taper from the coronal to apical [3].

To improve the effect of root canal therapy, various root canal preparation systems have been developed to maintain the original morphology of root canal anatomy (including using different instruments and cooperating with different solutions to eliminate the smear layer of the root canal wall). The results before and after the operation among groups were tested by the significance test, such as Mann–Whitney *U* test [3, 6, 7, 9]. With the development of medical imaging measurement technology such as CT, more and higher resolution image data can be collected, which boost more accurate instrument evaluation. *μ*CT is a nondestructive 3D imaging technology, which can clearly understand the internal microscopic structure of the sample. Hammad et al. [11] compared various instruments and considered that the root canal measurement results of the *μ*CT are consistent with the actual results. Kierklo et al. [6] evaluated the root canal preparation operation based on 2D data; that is, the cross-sectional images collected by the *μ*CT were compared before and after preparation. In [12], 3D models were reconstructed from images, and the volume and area of the model were used to evaluate the effect of preparation among different systems.

In the field of medical imaging, many studies of therapy evaluation are based on the idea of registration before comparison. As far as root canal models are concerned, there were many changes in the models before and after treatment, so the rigid registration method is unsuitable. The nonrigid registration method mainly includes two approaches based on geometric features or image statistics. Both of the two ideas need to extract features or statistical information from the model for registration. However, in most cases, it is difficult to obtain features accurately when the feature extraction is carried out between different models, or when the feature points are not distinct, and always accompanied by outliers. The outliners probably present immense barriers to the subsequent registration algorithm. Moreover, the geometric characteristics and the topology of the root canal model may change before and after preparation. It is also challenging to apply the idea of nonrigid registration to root canal preparation assessment.

**Figure 2:**
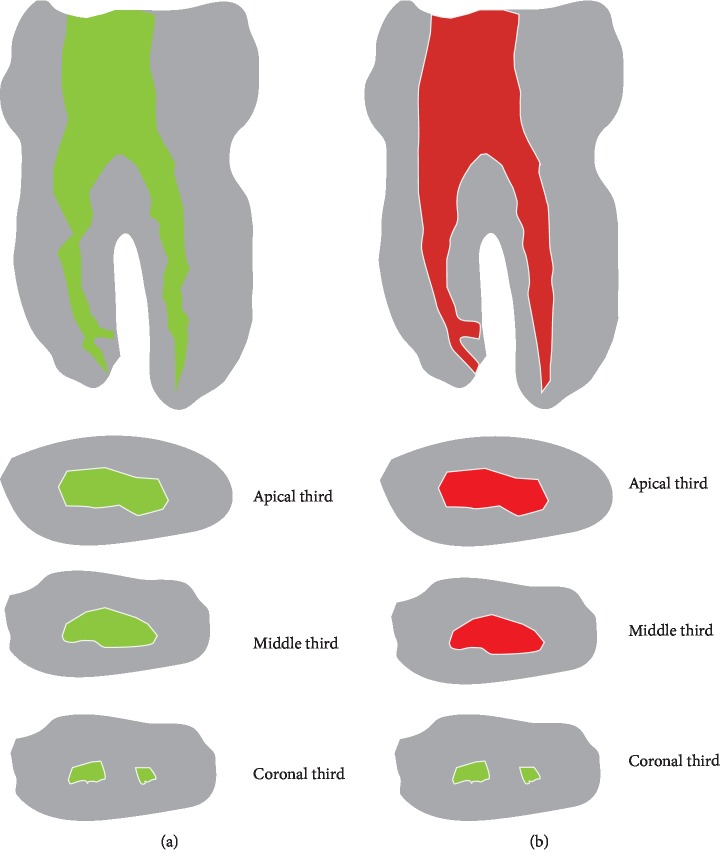
Calculating volume and area from slice images. (a) Before canal preparation. (b) After canal preparation.

Ørstavik [3] summarized the evaluation methods of root canal shaping ability of various instruments, including X-ray parallel projection, Bramante model, cross-sectional section method, CT, and *μ*CT methods. The ideas of these methods are to obtain the volume, surface area, and transportation of root canal axis before and after preparation by integrating the slice images [13, 14]. Specific methods are as follows:

1. Divide teeth with high *h* into *N* equal parts at the interval of *δ*=*h*/*N*, then get images {*I*_*i*_}_1_^*N*^
2. Measure the cavity area *S*_*i*_ and the perimeter of the cavity of the root canal *l*_*i*_ in *I*_*i*_
3. Calculate the total surface area with *S*=∑_*i*=1_^*N*−1^ *δl*_*i*_
4. Calculate volume with *V*=∑_*i*=1_^*N*^ *δS*_*i*_
5. The deviation of the root canal center and the average distance of each cross-sectional cavity are calculated, respectively, after registration, as shown in Figure 2

**Figure 3:**
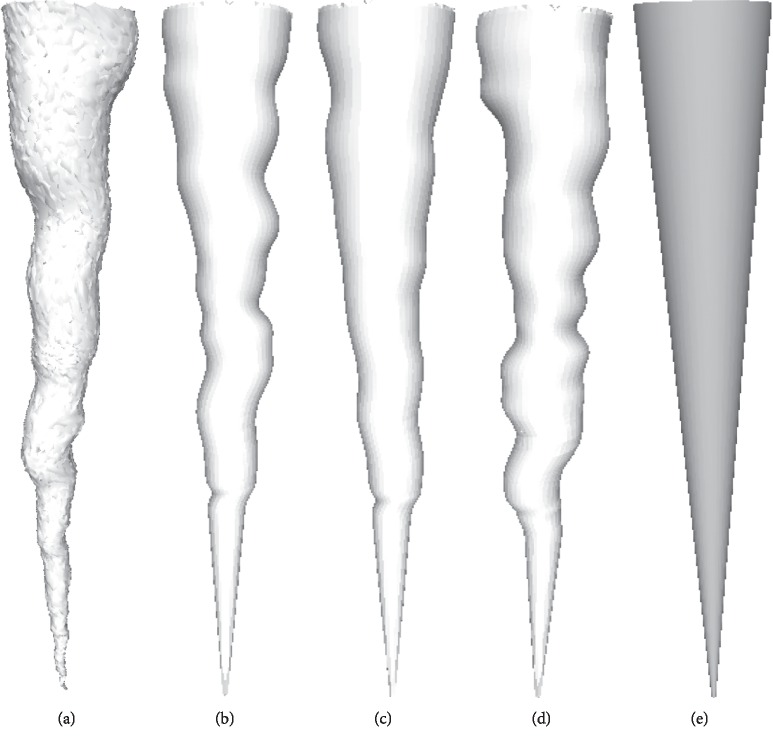
Five shapes with the same volume (intuitively, if shapes (a) to (d) were sorted from small to large in order of shape differences from the shape (e), the result is (c), (b), (a), and (d)).

Existing methods of root canal preparation evaluation have obvious drawbacks. First, the differences in permeability and taper of different root canals were submerged behind a single geometric quantity, which cannot distinguish the root canals with similar volume and significant shape difference (shown in Figure 3). Second, the overall geometry and complex topological structure of root canals are not taken into account. Third, the cavity morphology before and after treatment is often not considered. A large extent of transformation (irregular to ellipsoid-like) occurs, which may cause intolerable errors in both rigid and nonrigid registration algorithms.

Every root canal has its own individual shape. Accurate knowledge of the canal anatomy is a prerequisite for effective endodontic treatment [15, 16]. At present, the evaluation of the various instruments in root canal therapy is generally based on reexamining the volume percentage of the void (POV) of the root canal. This method typically requires a more extended period and cost. Therefore, by measuring the model of root canal preparation, defining the “distance” between the shapes and calculating the distance between the current root canal and the idealized root canal according to the criteria conducive to the subsequent root canal obturation, it will be helpful to improve the efficiency of this work.

**Figure 4:**
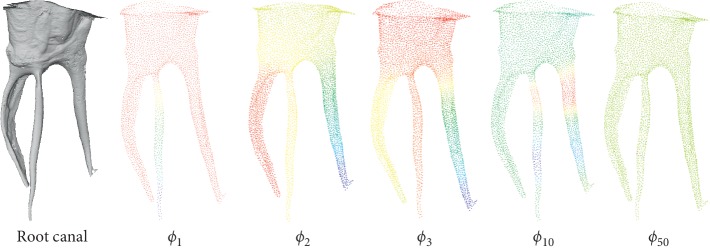
Root canal and its eigenfunctions from the Laplace–Beltrami spectrum.

For the first time in [17], the method of studying geometric shape based on the spectrum was proposed. The paper answered the question of how much about the shape can be inferred from the knowledge of its geometry spectrum. By calculating the shape spectrum {*λ*_*i*_} and its eigenfunction {*ϕ*_*i*_} as shown in Figure 4, the shape surface can be uniquely determined and vice versa in ordinary cases.

In recent years, with the development of 4G/5G technology and CT technology, as well as the unprecedented expansion of the accuracy and breadth of medical 3D data acquisition, more and more researchers pay attention to the massive data of medical geometry. On the contrary, the shape analysis paradigm, theory, and practice based on the geometric spectrum have gradually become a research hotspot [18–21]. Based on the works, the author proposed a novel method based on the geometric spectrum for root canal preparation treatment.

The spectrum of differential operator contains rich geometric and topological information of the shape, including curvature, area, and genus. The LBO spectrum and Steklov spectrum have the characteristics of independent of the coordinate system and scale. Although in rare high-dimensional manifolds, there may be different shapes sharing the same spectrum. However, in low-dimensional manifold cases (such as root canals), the geometry of the shape can be uniquely determined by its spectrum. The traditional shape spectrum is based on the intrinsic description of the Gauss curvature, which has an insufficient perception of concavity, convexity, and volume of shape, while the application of shape-based extrinsic geometric spectrum, such as Steklov spectrum, is increasing. It has gradually become a research hotspot [22–26].

In this work, we consider the problem of the root canal shape evaluation and investigate its relevance in a selection of literature from medical image and graphics. We propose a shape-aware evaluation method which is a precise evaluator for comparing different root canal preparation systems. Our key contributions can be summarized as follows:

  Firstly, despite being highly nonlinear and hard to compute, we indicate for the first time that the mapping between a 3D model of the root canal and its Steklov spectrum is addressable with modern mathematical tools
  Secondly, for the first time, an evaluation method of root canal shape based on an ideal root canal model is proposed, and an algorithm for generating an ideal root canal model is given
  At last, we evaluate the root canal preparation effect by defining the reasonable distance depending on the histogram of the Gaussian curvature and the Steklov spectrum of root canal models

## 2. Related Research

In recent years, more and more attention has been paid to the evaluation method of therapy based on 3D model reconstruction from data collected by *μ*CT. The high-resolution characteristics of *μ*CT make it widely used in small-scale medical in vivo research. In [27], synchrotron radiation microcomputed tomography (SR*μ*CT) was evaluated concerning the 3D morphology of the rat spinal cord microvasculature. In [28], Wang et al. used parallel programming techniques to accelerate existing image reconstruction algorithms for 3D optoacoustic tomography. In [29], Kobayashi-Velasco et al. compared cone-beam computed tomography (CBCT) with periapical radiograph (PR) in the diagnosis of alveolar and root fractures.

Also, more and more applications of *μ*CT in the evaluation of root canal therapy have been made. The purpose of root canal preparation is to clear the infected root canal anatomical space. Under ideal conditions, on the premise of keeping the centerline unchanged, the inner wall of the root canal should all be processed, and a better taper can be obtained to facilitate subsequent obturation. According to the different geometry of root canal preparation, the evaluation methods of root canal preparation can be divided into two categories.

The first category focuses on volume and area changes before and after root canal preparation. In [10], the root canal was segmented into the coronal third, middle third, and apical third. Scanning electron microscopy (SEM) was used to observe the removal of the smear layer; then the images before and after root canal preparation were calculated and compared, which was used to evaluate the therapy. In [4], the geometry of the root canals was described by three different mathematical models: the elliptical model, the 1-ring model, and the 3-ring model, and then a method for analyzing the geometric characteristics of root canals was realized. In [30], Berutti et al. compared the canal curvature and axis transportation after instrumentation with three different systems. In [31], the critical section images of the coronal third, middle third, and apical third before and after root canal preparation were analyzed and compared by several nickel-titanium systems. Anbu et al. [32] observed the efficacy of various technique to fill root canals using spiral computed tomography (SCT). Orhan et al. [2] used both *μ*CT and nano-CT to measure and analyze the volume ratio of filling voids at coronal third, middle third, and apical third and recommend that a voxel size of 11.2 *μ*m can be used as a reliable cutoff value in *μ*CT imaging.

The second category of the evaluation method focuses on the transportation of spatial centerline and the balance of periodontal dentin thickness. In [9, 16], root canal deviation and central positioning (or transportation) ability were considered to be essential parameters for evaluating the quality of canal preparation. In [33, 34], the thickness of the mesial and distal root canal wall was scanned and measured, respectively, and then the displacement and axial centrality of root canals in each group were calculated by using Gambill's formula. In in vivo cases, the coordinate systems of canal measured before and after the operation are not consistent in general. How to register the two root canals remains a problem to be solved. Because of the particularity of teeth, it is not feasible to use registration points in industrial scenes for registration.

There are two shortcomings in the above two methods: it significantly relies on knowledge and experience manipulators on understanding the geometry of the root canal. The accuracy and automation of the process are not high; the other is that the evaluation based on local sampling lacks a global shape assessment method suitable for subsequent obturation, so it is not conducive to accurately evaluate the quality of root canal preparation of various instruments.

3D shape comparison is a hotspot in the field of geometric analysis. In the field of manufacturing, it is often necessary to evaluate the accuracy of parts processing by shape comparison, such as blades and special-shaped bolts. In the medical field, the manufacture of porcelain fused to metal teeth and artificial bones also need to evaluate the difference of shape. In online massive shape retrieval scenarios, it is necessary to quickly match one or some kinds of shape according to certain retrieval conditions. In the interactive game industry, it is required to cluster and understand facial expressions. In [35], the mapping between two surfaces is expressed by the linear function map, and the difference between different shapes is evaluated by defining reference shapes. Fish et al. [36] introduced a meta-representation that represents the essence of a family of shapes. Handels and Hacker [37] extended the volume-based representation of organs to enable the 3D visualization of organs' shape variability in the atlas. In [38], a functional map between two shapes based on the multiscale Laplace–Beltrami operator (LBO) shape descriptor was proposed, and the difference between shapes was evaluated by the basis function family of the spectrum to avoid the NP-hard problem of the registration algorithm. In [39], the comparative shapes were embedded in canonical domains and evaluated.

**Figure 5:**
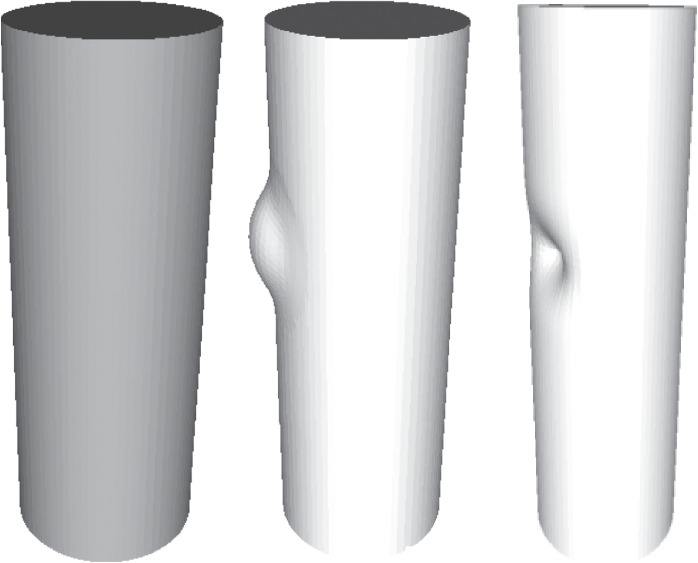
Inward and outward bumps on the cylinders.

Shape comparison methods can be categorized into three types: first, the corresponding relationship between shapes is established, and then the geometric comparison is made; the other is to compress the geometric and topological information of three-dimensional image into a scalar, i.e., shape similarity, by using some global or local shape descriptors; and the third is to evaluate the shape difference by defining a specific norm from the global perspective, such as [40]. These shape descriptors are all shape descriptors based on intrinsic geometry or geodesic equidistant transformation invariant. However, the intrinsic description of shape, such as the LBO spectrum, is unable to distinguish concave and convex shapes (Figure 5). The conicity, concavity, and convexity of root canal shape and the overall permeability are the key factors to evaluate the effect of treatment. Therefore, the extrinsic shape descriptor, which can capture volume difference, is suitable in the scenario. According to [41, 42], the spectrum of the Steklov operator mapping the Dirichlet boundary to Neumann boundary is precisely extrinsic.

The problem of calculating the spectrum of the Steklov operator of shape is generally transformed to solving the boundary value problem of the partial differential equation (PDE). Because of the complexity and discontinuity of the boundary, it is almost impossible to obtain the analytic solution. Therefore, finding the weak solution of the equation is the only feasible choice. Solving the PDE on the discrete point cloud model by the spectral method is an effective way to improve the accuracy and convergence speed. The basic idea is to map the original PDE to an integral equation. By using the orthogonal and recursive formulas of the basis function, the weak solution problem of PDE is simplified to an integral equation and then converted to the eigenvalue problem of linear equations [43]. Therefore, the main obstacle is to select a fast convergent of the basis function family to calculate the shape spectrum for the root canal as a specific boundary condition.

The main contribution of this study is to overcome the shortcomings of the previous methods of evaluation of root canal preparation by surface area, volume, or eccentricity thickness of the central line. Based on strict mathematical theories, the proposed methods for comparing shape differences are given in terms of conicity, permeability, and smoothness of the whole root canal. This method does not require the establishment of calibration points for the shape of the root canal. This thought avoids the precondition that two shapes must share the same topological structure in previous algorithms and is very suitable for the shape comparison of the root canal. It also breaks through the problems of different CT sampling rates and inconsistent coordinates of in vivo data acquisition and evaluates root canal preparation more accurately and comprehensively.

## 3. Theoretical Analysis

### 3.1. Main Idea

Thanks to the excellent characteristics of the Steklov spectrum in describing concave and convex shapes and volume changes and its robustness in the calculation, the authors propose an algorithm of root canal treatment evaluation based on the shape difference of the Steklov spectrum. The overall idea is as follows:

1. Firstly, the 3D models before and after root canal preparation *M*_0_ and *M*_1_ were acquired by *μ*CT.
2. To synthesize the changes of a cross section of *M*_0_ and *M*_1_ at the same height, the key cross section was selected, and the ideal root canal model ${\tilde{M}}_{1}$ was generated by lofting.
3. Calculate the Steklov shape spectrum of *M*_1_ and ${\tilde{M}}_{1}$, and then get the eigenvalues {*λ*_*i*_} and {*μ*_*j*_}.
4. Define the spectral distance Steklov spectrum discrete (SSD) to evaluate the difference. The distance is used to measure the shape difference between *M*_1_ and ${\tilde{M}}_{1}$ so as to evaluate the quality of root canal preparation.

### 3.2. Mathematical Model of the Steklov Spectrum

In the three-dimensional Euclidean space, the Steklov eigenvalue problem is defined as follows.

Let *Ω* ∈ ℝ^3^ be a compact Riemannian manifold, whose boundary *Σ* = ∂*Ω* satisfies the Lipschitz condition. The Steklov equation is used to find the harmonic function *u*(*x*) and real number *λ* ≠ 0. The Steklov problem on *Ω* is

(1) $$\begin{array}{c} \left\{\begin{array}{cc} \Delta u\left(x\right)=0, & x\in \Omega ,\\ \frac{\partial u\left(x\right)}{\partial \overset{⟶}{n}}=\lambda u\left(x\right), & x\in \Sigma , \end{array}\right. \end{array}$$

where Δ = ∑_*i*=1_^3^∂^2^/∂*x*_*i*_^2^ is the Laplace operator on functions on *Ω* and $\partial u\left(x\right)/\partial \overset{⟶}{n}$ is the outward derivative along *Σ*. In the second equation in equation (1), it can be interpreted as an eigenvalue problem that transforms the Dirichlet boundary condition into the Neumann boundary condition. It maps function *f* ∈ *C* ^*∞*^(*Σ*) to *𝒟f* = ∂_*n*_(*Hf*) ∈ *C* ^*∞*^(*Ω*):

(2) $$\begin{array}{c} D:f⟶\partial_{n}\left(Hf\right), \end{array}$$

where *𝒟* is the elliptic pseudodifferential operator of order one. *Hf* is the harmonic extension of *f* on *Ω* and extends smoothly from the boundary to the interior of *Ω* [44, 45]. The eigenfunctions {*ϕ*_1_, *ϕ*_2_,…, } corresponding to the eigenvalues constitutes the orthogonal basis in *L* ^2^(∑) space. The trace of the eigen matrix formed by {*ϕ*_*i*_} is closely related to the intrinsic geometry and the extrinsic geometry of the shape itself.

Let Tr(*e* ^−*t𝒟*^) be the heat kernel of the operator *e* ^−*t𝒟*^ : *f*⟶*u* for the heat equation ∂_*t*_ *u*+*Pu*=0, when *t*⟶0^+^, and there is an asymptotic expansion of Tr(*e* ^−*t𝒟*^) [46]:

(3) $$\begin{array}{c} h\left(x,t\right)≔\text{Tr}\left(e^{-tD}\right)=\int_{\Sigma }e^{-tD}\left(x,x\right)dx∼\overset{\infty }{\underset{k=0}{\sum }}a_{k}\left(x\right)t^{-n+1+k}+\overset{\infty }{\underset{l=1}{\sum }}b_{l}\left(x\right)t^{l}\log\;t. \end{array}$$

The coefficients *a*_0_, *a*_1_,…, *a*_*n*−1_, as well as *b*_0_, *b*_1_,…, *b*_*n*−1_, are the Steklov heat invariants which are determined by the Steklov spectrum. According to [26], when *n*=3 and *Ω* ∈ *ℝ* ^3^,

(4) $$\begin{array}{c} a_{0}\left(x\right)=\frac{1}{2\pi }, \end{array}$$

(5) $$\begin{array}{c} a_{1}\left(x\right)=\frac{K_{H}\left(x\right)}{4\pi }, \end{array}$$

where *K*_*H*_(*x*) is the mean curvature at *x*.

(6) $$\begin{array}{c} a_{2}\left(x\right)=\frac{1}{16\pi }\left(K_{H}\left(x\right)+\frac{R_{M}\left(x\right)}{6}\right), \end{array}$$

where *R*_*M*_(*x*) is the Gaussian curvature of boundary Σ.

From equations (3) to (6), it can be seen that the mean curvatures reflecting the intrinsic geometry and the Gaussian curvature of the extrinsic geometry are determined by the Steklov spectrum.

In summary, by calculating the eigenvalues and eigenfunctions of the Steklov operators, the intrinsic geometry and extrinsic geometry of shape can be transformed into the spectrum space for research, and then shape difference evaluation can be carried out. Our thought is based on PDE, which obtains the whole geometric structure from the boundary, which is a powerful mathematical tool for medical CT imaging and geological exploration imaging [47].

According to the literature [48], the Steklov spectrum and LBO spectrum of shape are invariant in isometric transformation. Moreover, it has strong robustness for model downsampling, and even nonuniform sampling has better immune effect for topological noise. What is more, the conjugate gradient descent method can be used to solve the above spectrum problem, and the running speed of the algorithm does not depend significantly on the number of edges and faces. The Steklov spectrum has good accuracy even for degenerated irregular meshes. Therefore, it is very beneficial to realize the fast evaluation of root canal shape difference.

### 3.3. Discussion on the Numerical Solution of the Steklov Spectrum

The numerical solution of PDE boundary value problems for high-dimensional manifolds, and two-manifold with higher genus often falls into local minima or diverges, leading to calculation failure. A series of thresholds or carefully adjusted penalty functions were often needed to intervene manually. The 3D topology and geometry of root canals are relatively fixed (tree-shaped cavity), gradually thinning from top to bottom. Therefore, it is possible to avoid the shortcomings of the general algorithm and improve the automaticity and accuracy of the algorithm by thoroughly combining this priori knowledge. Thus, the design of a particular shape difference evaluation algorithm based on relatively fixed root canal topology will be based on the conicity, permeability, and smoothness of the whole root canal shape. The shortcomings of the previous local and single geometric evaluation method were broken through to provide a more scientific and comprehensive assessment method for root canal preparation effect. The calculation method is deduced as follows.

The numerical solution is based on potential theory. The potential theory is a robust basis for solving mathematical and physical equations by using the integral equation method. By using the linear superposition principle and fundamental solutions, various forms of integral expressions are obtained. Then, by solving the boundary, the purpose of solving the definite solution problem of the differential equation corresponding to the physical problem is achieved.

In this paper, the boundary element method is used to solve the Steklov eigenvalue problem. This method is different from the finite element method, the variational method, and the radial basis function method. It has the characteristics of simplicity, fast convergence, and high accuracy. It is very suitable for the case of complex internal structure and gridding [49]. The basic idea of the boundary element method is that first, the differential equation is transformed into a boundary integral equation. After that, the discrete quadrature method is used for numerical calculation. In solving the discrete matrix elements of the integral equation, the solution needs to be obtained by a singular integral. In this paper, after derivation, the asymptotic expansion of the odd order can be obtained. When the accuracy can be achieved, the convergence speed of the eigenvalue problem can be significantly improved. At the same time, a posterior error estimate can be obtained based on residual estimation [50].

According to the potential theory of the differential equation, when *y* is a nonsingular point on Σ, the Steklov eigenvalue problem can be converted to the following integral equation with the Green formula [51]:

(7) $$\begin{array}{c} \frac{1}{2}u\left(x\right)=-\int_{\Sigma }\frac{\partial h\left(x,y\right)}{\partial n_{y}}u\left(y\right)ds_{y}+\int_{\Sigma }h\left(x,y\right)v\left(y\right)ds_{y}, \end{array}$$

where *v*(*x*)=∂*u*(*x*)/∂*n*_*x*_ and *U* ^*∗*^(*y*, *x*)=1/(4*π*|*x* − *y*|) is the fundamental solution of the following equation:

(8) $$\begin{array}{c} \left(L_{y}G\right)\left(x,y\right)=\delta_{0}\left(y-x\right),\;\text{for }x,y\in {\mathbb{R}}^{3}. \end{array}$$

Thus, the Steklov eigenvalue problem is transformed into the boundary element numerical calculation problem. The first term on the right-hand side of equation (7) is the double-layer potential, the second term is the single-layer potential, and the corresponding density functions are −*u*(*y*) and *v*(*x*).

According to the continuity of single-layer potential on the boundary, equation (7) still holds for *x* ∈ Σ. According to the discontinuity of the single-layer potential derivative and the nature of double-layer potential, the following relations can be obtained:

(9) $$\begin{array}{c} \frac{\partial u}{\partial n}=V^{-1}\left(\lambda I+K\right)u, \end{array}$$

where *V* : *H* ^1/2^(Σ)⟶*H* ^1/2^(Σ) is the single-layer potential operator.

(10) $$\begin{array}{c} \left(Vw\right)\left(x\right)≔\int_{\Sigma }U^{*}\left(x,y\right)w\left(y\right)ds_{y}, \end{array}$$

where *U* ^*∗*^(*y*, *x*)=1/(4*π*|*x* − *y*|) is the fundamental solution of Δ*u*(*x*)=0. The integral operator *V* maps the density function *w*(*x*) to the potential distribution on Σ.

*K* is the double-layer potential as follows:

(11) $$\begin{array}{c} u\left(x\right)=\left(Kv\right)\left(x\right)≔\int_{y\in \Sigma :\left|y-x\right|\geq \varepsilon }\frac{\partial U^{*}\left(x,y\right)}{\partial n_{y}}v\left(y\right)ds_{y}. \end{array}$$

Since the right-hand side of equation (9) is not necessarily a positive definite matrix after being discretized into a matrix, the conjugate gradient method cannot be used to calculate its eigenvalues. According to [51], equation (9) can be transformed into another eigenvalue problem with the same solution through the Calderón projection:

(12) $$\begin{array}{c} \frac{\partial u\left(x\right)}{\partial n}=\left[D+\left(\sigma \left(x\right)I+K^{*}\right)V^{-1}\left(\sigma \left(x\right)I+K\right)\right]u\left(x\right)=\lambda u\left(x\right),\;\text{for }x\in \Sigma , \end{array}$$

(13) $$\begin{array}{c} \sigma \left(x\right)≔\underset{\varepsilon ⟶\infty }{\lim}\frac{1}{4\pi \varepsilon^{2}}\int_{y\in \Omega :\left|y-x\right|=\varepsilon }ds_{y},\;\text{for }x\in \Sigma , \end{array}$$

for almost *x* ∈ *Σ*, *σ*(*x*) = 0.5 [51].

In equation (12), *D* is a hypersingular operator which is defined as

(14) $$\begin{array}{c} \left(Dv\right)\left(x\right)≔-\int_{\Sigma }\frac{\partial^{2}U^{*}\left(x,y\right)}{\partial n_{x}\partial n_{y}}v\left(y\right)ds_{y}. \end{array}$$

*K* ^*∗*^ : *H* ^−1/2^(Σ)⟶*H* ^−1/2^(Σ) is the adjoint double-layer potential, which is defined as the conormal derivative of *V*:

(15) $$\begin{array}{c} \left(K^{*}w\right)\left(x\right)=\int_{\Sigma }\frac{\partial U^{*}\left(x,y\right)}{\partial n_{x}}w\left(y\right)ds_{y}. \end{array}$$

The potential functions discussed above are integral operators, which are defined in both continuous space and infinite-dimensional function space. However, the root canal model is discretized, so these boundary operators should be further discretized into corresponding matrices which is discussed in Section 4.

### 3.4. Constructing an Ideal Root Canal Model as a Benchmark

The ideal model serves as a benchmark for the evaluation of preparatory effect. For clearing the inner wall of the root canal, facilitating subsequent obturation and minimizing the transportation of centerline of the root canal, the prepared root canals should have the following criteria:

1. The central axis should be consistent with the original one
2. The new cross section should be enlarged evenly on the basis of the old one to ensure that all the inner walls are treated evenly
3. The volume of the cut inner cavity should be as small as possible on the premise of satisfying Step (2)
4. The transition between sections should be as smooth as possible

**Figure 6:**
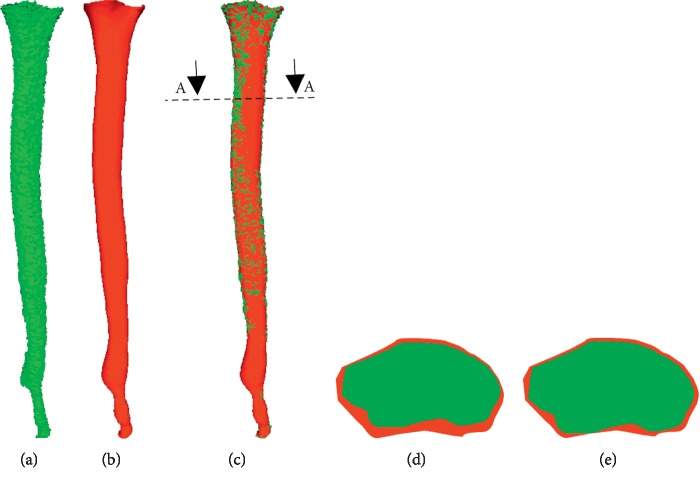
Comparison of two results before and after root canal preparation. (a) Before preparation. (b) After preparation. (c) Compound root canal. (d) Result I at A-A (5x). (e) Result II at A-A (5).

Based on the four criteria, the second result is better than the first result in Figures 6(d) and 6(e).

Since most of the sections are nearly circular, we propose an ideal algorithm of root canal model generation based on the ideal inner diameter. The constructing steps are as follows:

(1) Assuming that the height of the root canal is *h*, a root canal is divided into three segments: coronal, middle, and apical from top to bottom with heights *h*_*c*_, *h*_*m*_,  and *h*_*a*_, respectively. For the coronal segment, three sections are intercepted at three heights of (*h*_*c*_/3) − *a*, (*h*_*c*_/3) and (*h*_*c*_/3)+*a* (*a*=0.05*h*_*c*_). Also in the middle and apical segments, three sections each are intercepted in the same way as in coronal's. A total of three groups of 9 sections {*S*_*i*_^*c*^}_*i*=1_^3^, {*S*_*j*_^*m*^}_*j*=1_^3^,  and {*S*_*k*_^*a*^}_*k*=1_^3^ are obtained, and the set of boundary points of the sections are {**p**_*i*_^*c*^}_*i*=1_^3^, {**p**_*j*_^*m*^}_*j*=1_^3^,  and {**p**_*k*_^*a*^}_*k*=1_^3^, respectively.
(2) To calculate the ideal inner diameter of the section, the following formulas are used:
  (16) $$\begin{array}{c} r_{\text{ideal}}\left(\cdot \right)≔\sqrt{\frac{A\left(\cdot \right)}{\pi }}, \end{array}$$
  where *A*(·) represents the area of the given section.
(3) For each section, the average distance $\bar{d}\left(\cdot \right)$ between the actual boundary and the ideal boundary is calculated by using the following formula:
  (17) $$\begin{array}{c} \bar{d}\left(\cdot \right)≔\frac{\sum_{i=1}^{N}\left(\left\|p_{m}-c\left(\cdot \right)\right\|-r_{\text{ideal}}\left(\cdot \right)\right)}{N}, \end{array}$$
  where *p*_*i*_ denotes the *i*th boundary point of the current section and *c*(·) is the barycentric coordinates of the boundary points of each section. *N* represents the number of boundary points of the current section. For each segment of the root canal, the cross section with the minimum $\bar{d}\left(\cdot \right)$ is selected as the representative section of the segment. Obviously, when the section is circular, its $\bar{d}\left(\cdot \right)$ equals zero exactly.
(4) In Step (3), the critical cross sections *S* ^*c*^, *S* ^*m*^,  and *S* ^*a*^, of the three segments were obtained. The top section *S* ^*t*^ and the bottom section *S* ^*b*^ of the root canal can be achieved from the original model. The ideal root canal model can be generated by lofting in the order of *S* ^*t*^, *S* ^*c*^, *S* ^*m*^, *S* ^*a*^, and *S* ^*b*^.

## 4. Implementation of Algorithms

### 4.1. Spectrum Numerical Solution Algorithm

The potential operator is defined in infinite-dimensional function space, and the actual root canal model is a triangular mesh composed of finite points. In this paper, the Galerkin method is used to convert the continuous equation to weak form. Specifically, by defining the basis function, some constraints are imposed on the original function space [42]: finding *ϕ* ∈ *ℍ* ^*d*^(Σ) in function space *ℍ*, such as that for all *u* ∈ *ℍ* ^*d*^(Σ), *a*(*ϕ*, *u*)=*f*(*u*) ∈ *ℝ*, where *a*(·, ·) represents a bilinear form, and it is also a bounded linear function. Therefore, for the weak form of single-layer *u*_*n*_, it is expressed by a set of basis function {*e*_1_, *e*_2_,…, *e*_*n*_}:

(18) $$\begin{array}{c} u_{n}=\overset{n}{\underset{j=1}{\sum }}a\left(e_{i},e_{j}\right)u_{j}. \end{array}$$

Thus, the original integral problem on the whole boundary is transformed into the issue of solving weak form linear equations.

Let the discretized root canal model be *M*=(V,E,F). Substitute equation (18) into integral equations (equations (10), (11), (14), and (15)). These integral equations will be discretized into summation form and can be expressed in matrix form eventually.

Any piecewise linear hat function *ϕ*_*i*_ : Σ⟶*ℝ* on *M* equals one at their associated vertex and zero at all other vertices and is orthogonal at different subscripts. Therefore, any square integrable function $\tilde{u}$ on *M* can be expressed as

(19) $$\begin{array}{c} \tilde{u}=\underset{i}{\sum }c_{i}\phi_{i},\\ c_{i}=\int_{\Sigma }\langle \phi_{i},u\rangle dx,\;c_{i}\in \mathbb{R}. \end{array}$$

Assume that *w*(*x*) ∈ Σ is the solution of

(20) $$\begin{array}{c} Vw\left(x\right)=f\left(x\right). \end{array}$$

Then, the weak form of equation (20) is

(21) $$\begin{array}{c} {\langle \phi ,Vw\rangle }_{\Sigma }={\langle \phi ,f\rangle }_{\Sigma }. \end{array}$$

After *Σ* is discretized into the mesh *M*, *w* and *f* are also discretized into vectors , so the following linear system can be obtained:

(22) $$\begin{array}{c} Vw=Gf\text{,} \end{array}$$

where **V** = {v_*ij*_},**G** = {g_*ij*_}, and

(23) $$\begin{array}{c} v_{ij}=\underset{T_{1}\in T\left(i\right)}{\sum }\underset{T_{2}\in T\left(j\right)}{\sum }\iint_{T_{1}\times T_{2}}U^{*}\left(x,y\right)\phi_{i}\left(x\right)\phi_{j}\left(x\right)ds_{x}ds_{y},\\ g_{ij}=\underset{T_{1}\in T\left(i\right)}{\sum }\int_{T\left(i\right)}\phi_{i}\left(x\right)\phi_{j}\left(x\right)ds_{x}. \end{array}$$

Similarly, the potential integral equations in equation (12) can be listed as a series of linear systems, namely,

(24) $$\begin{array}{c} D⟶D=\left\{d_{ij}\right\},\\ K⟶K=\left\{k_{ij}\right\},\\ K^{*}⟶K^{*}=\left\{k_{ij}^{*}\right\}\text{,} \end{array}$$

where *d*_*ij*_ is defined as

(25) $$\begin{array}{c} d_{ij}=\frac{1}{4\pi }\underset{T_{1}\in A\left(i\right)}{\sum }\underset{T_{2}\in A\left(j\right)}{\sum }\iint_{T_{1}\times T_{2}}\left[\frac{3\left(y-x,n_{x}\right)\left(y-x,n_{y}\right)}{{\left|x-y\right|}^{5}}-\frac{\left(n_{x},n_{y}\right)}{{\left|x-y\right|}^{3}}\right]\phi_{i}\left(x\right)\phi_{j}\left(x\right)ds_{y}, \end{array}$$

and *n*_*x*_ and *n*_*y*_ denote the normal directions.

Also in equation (24), *k*_*ij*_ is defined as

(26) $$\begin{array}{c} k_{ij}=\frac{1}{4\pi }\underset{T_{1}\in A\left(i\right)}{\sum }\underset{T_{2}\in A\left(j\right)}{\sum }\iint_{T_{1}\times T_{2}}\left[\frac{3\left(x-y,n_{y}\right)}{{\left|x-y\right|}^{3}}\right]\phi_{i}\left(x\right)\phi_{j}\left(x\right)ds_{y}, \end{array}$$

where *k*_*ij*_^*∗*^ is defined as

(27) $$\begin{array}{c} k_{ij}^{*}=\frac{1}{4\pi }\underset{T_{1}\in A\left(i\right)}{\sum }\underset{T_{2}\in A\left(j\right)}{\sum }\int_{T_{1}\times T_{2}}\left[\frac{3\left(y-x,n_{x}\right)}{{\left|x-y\right|}^{3}}\right]\phi_{i}\left(x\right)\phi_{j}\left(x\right)ds_{y}. \end{array}$$

From the above discussion, we then obtain that the Steklov eigenvalue problem on discrete meshes is

(28) $$\begin{array}{c} \left[D+\left(\mathrm{0.5}I+K^{*}\right)V^{-1}\left(\mathrm{0.5}I+K\right)\right]u=\lambda Gu\text{.} \end{array}$$

The matrices **D**, **K**, **K** ^*∗*^, **V**,  and **G** in equation (28) are symmetrical, and the summation and inverse matrices are symmetrical and positive definite matrices, which can be solved by using the conjugate gradient algorithm. Firstly, we rewrite equation (28) as follows:

(29) $$\begin{array}{c} \tilde{A}\bar{U}=0. \end{array}$$

In this paper, the PCG method is used to solve equation (29). The main idea is to transform the linear system (29) into equivalent functional optimization problems. In view of the convergence of the steepest descent method and the insufficiency of the conjugate gradient method depending on the distribution of eigenvalues, the incomplete Cholesky decomposition conjugate gradient (ICCG) method not only keeps the matrix sparse but also makes the preoptimized matrix approximate identity matrix. Therefore, the method accelerates the iteration process [52].

Assume that **L** ^*T*^ **L** is an approximate decomposition of **A**:

(30) $$\begin{array}{c} \tilde{A}=L^{T}L+R, \end{array}$$

where **R** is a residual matrix and can be used to control the sparsity of **L**. Since the elements of **L** should ensure that the preoptimal matrix is effective, **L** ^*T*^ **L** is an approximate decomposition of **A**. This paper adopts the following strategies.

Referring to [53], if the element ${\tilde{a}}_{ij}$ of $\overset{˜}{A}$ is not zero, then the element *l*_*ij*_ of **L** is calculated with the complete Cholesky decomposition formula. Otherwise, if ${\tilde{a}}_{ij}=0$, then let *l*_*ij*_ = 0. After finding the incomplete decomposition of $\tilde{A}$ (let **L** be nonsingular), $\tilde{A}=L^{T}L$ can be taken as the preoptimal matrix. Since **L** is not singular, $\overset{˜}{A}$ is symmetrically positive definite. Thus, the process of solving the linear system $\tilde{A}\bar{U}=0$ can be converted to solving the following two triangular matrix linear systems:

(31) $$\begin{array}{c} L\tilde{y}=0,\\ L^{T}\tilde{z}=\tilde{y}. \end{array}$$

In this work, heuristic strategies were used to detecting the convergence rate at different resolutions to determine the optimal sparse degree of the direction. The specific algorithm is as follows.

### 4.2. Root Canal Shape Evaluation Based on the Spectral Difference

In many kinds of literature, shape difference evaluation based on landmark points is the primary method, including geodesic distance based on point pairs and spectral distance based on point pairs. These methods require that there are well-matched points between two models to be compared, and registration is needed before comparison. Most of the shapes before and after root canal treatment have nonrigid deformation. It is almost impossible to find the corresponding points in the shape of the operation, so the above methods are not suitable for the problems studied in this paper.

In the related literature of shape differences, Ovsjanikov et al. [38] presented a novel representation of maps between pairs of shapes, and an energy measuring distortions induced by a map was defined on the function space to measure the difference of shapes. Huang et al. [54] proposed a pipeline for constructing an area-based shape difference operator on point clouds and showed numerically that the results are robust and informative.

Suppose the spectrum extracted from the two root canal models is ***λ***={*λ*_*i*_} and ***μ***={*μ*_*i*_}. A naive difference evaluation method is to calculate the Euclidean distance between ***λ*** and ***μ*** as

(32) $$\begin{array}{c} d_{s}\left(M,\tilde{M}\right)={\left\|\lambda \;-\;\mu \right\|}_{2}. \end{array}$$

However, the influence of different eigenvalues on shape is different since the first dozens of eigenvalues determined the shape.

Kac [17] proposed the spectral distance weighted by *i* ^−1^ but did not consider the influence factors of the regional characteristics of a specific shape.

**Figure 7:**
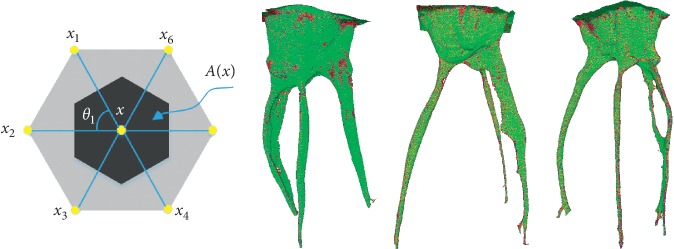
An example of calculating the Gauss curvature of the root canal model.

Based on the spectral theory, we know that the low-frequency eigenvalue reflects the global geometry (that is, the high-frequency signal is cancelled in a flat place) and the high-frequency eigenvalue reflects the local geometry (resonance occurs at the end of the shape). This principle was used in document [19, 21, 55, 56] to realize the interchange of gestures and details of multiple animated characters. Therefore, for each eigenvalue, its contribution to the difference between two shapes is proportional to the size of the variation range of the shape details. Intuitively, the curvature is the amount by which a geometric object such as a surface deviates from being a flat plane. The intensity of detail changes on the mesh can be quantified by the Gaussian curvature. The Gaussian curvature of the triangular mesh surface can be calculated by using the following formula:

(33) $$\begin{array}{c} K\left(x\right)=\frac{1}{A\left(x\right)}\left(2\pi -\underset{x_{i}\in N_{1}\left(x\right)}{\sum }\theta_{i}\right), \end{array}$$

where *x* ∈ **V** and *A*(*x*)is the area of the 1-ring region centered on *x*. *θ*_*i*_ is the angle of one of the adjacent triangles. Examples of the Gaussian curvature are shown in Figure 7.

Accordingly, we propose a spectral difference evaluation algorithm based on Gaussian curvature statistical distribution. Suppose the mesh model of a root canal is *M*=(V,E,F), *N*(*v*) represent the number vertex in V. We select the first *l* eigenvalues in the spectrum to evaluate the shape difference. The spectral difference evaluation result *d*(*M*, *N*) can be calculated by using Algorithm 1.

Through the above algorithm, we can quantify the weight of the difference between two spectrums in the overall shape difference comprehensively. Therefore, we can get better experimental results.

## 5. Experiment and Results

Traditional evaluation indicators for the effectiveness of systems include volume and area difference, as well as the transportation of the root canal. In this paper, the conicity and permeability of root canals are evaluated from the spectral distance between shapes. To access the superiority of the algorithm, the accuracy and the robustness of the algorithm are evaluated.

### 5.1. Programming

This paper mainly deals with the numerical solution of the integral equation. The most crucial problem is to find the eigenvalues of a matrix, i.e., to solve large-scale sparse linear system. Therefore, we choose the popular eigen algorithm library to calculate. It provides a series of built-in solver libraries and encapsulates some external solver libraries, including LLT and LDLT used in this paper. The algorithm proposed in this paper was implemented through C++ programming with the hardware and software shown in Table 1.

### 5.2. Accuracy Evaluation

#### 5.2.1. Comparison with LBO-Based Method

The Laplace–Beltrami operator is an intrinsic invariant operator whose spectrum also reflects the geometric and topological characteristics of some shapes, so it is often used for shape difference comparison, such as [57]. Most of the models processed by previous works are mesh models with low genus or high regularity. However, the mesh generated from the point cloud of the root canal obtained from *μ*CT is often of poor quality. Therefore, for some spectral methods with low robustness, there may be more problems in the aspect of accuracy.

**Figure 8:**
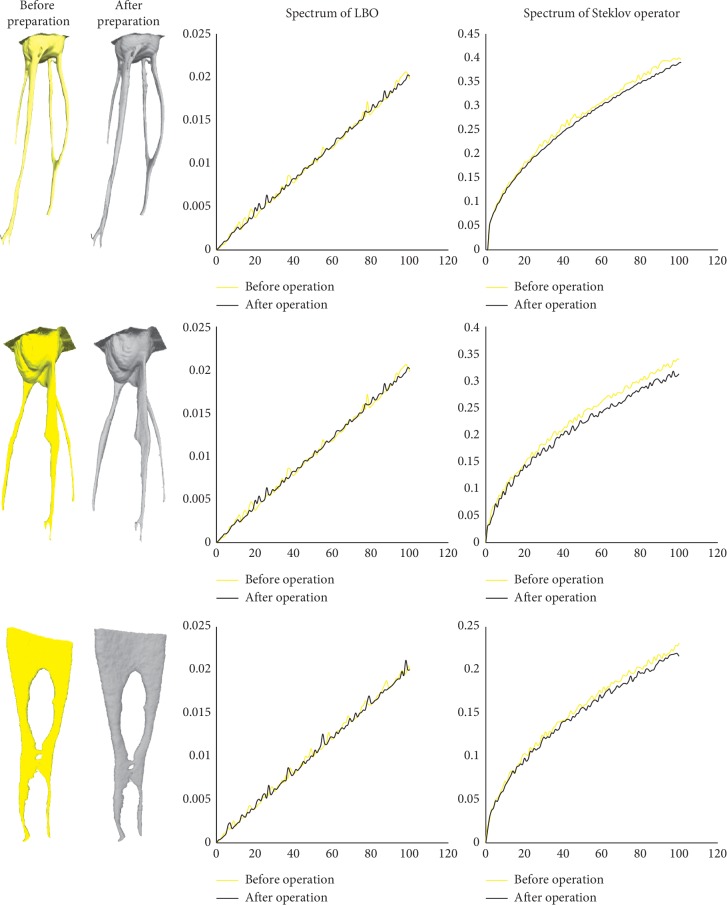
Comparison of the Steklov spectrum and LBO spectrum of the root canal model. Yellow indicates the original root canal, and grey indicates the root canal after preparation in all graphs and charts.

In this paper, the Steklov spectrum method and LBO spectrum method are used to calculate three groups of root canal models, respectively, and the results are shown in Figure 8. Our algorithm has an apparent distinction between the apparent uneven root canal shape, the rough root canal, and the different topology, while the performance of the LBO method is not ideal. Therefore, we infer that our algorithm is more suitable for the root canal model with complex geometry and topology in these cases.

#### 5.2.2. Comparison with Volume/Area Evaluation Method

Our method is to compare the prepared root canal with the idealized root canal and then calculate the overall geometric structure and topology. Shape difference based on the extrinsic spectrum accurately perceives the concavity and convexity of the shape. So our method is volume aware, area aware, and slice aware in real sense.

To evaluate the comparative effect of SSD, we infer that the best root canal shape is the smallest distance from the ideal root canal shape in theory, and the best therapy result is obtained. In [32], spiral computerized tomography (SCT) was reported to be a useful tool in various *in vivo* and laboratory studies. It was concluded that with SCT, three-dimensional volume measurements are possible without sectioning specimens, thus avoiding loss of material.

Specifically, the comparative method is as follows: A total of 60 extracted first molars were allocated into three groups randomly. Each group was treated with one instrument, respectively. The effect of root canal preparation was evaluated by the literature [2, 14, 58], which was based on the percentage of void (POV). The lower the POV, the better the obturation effect.

The aim of this comparison is to assess the correctness of the volume and area evaluation method and our method. Statistical analysis was performed with ANOVA (Scheffé tests).

The results of experiments summarized in Table 2 show that there is no significant difference among the three groups of results obtained by the volume-based discriminant method, and the third group which is considered to be the best one is not consistent with the ground truth evaluation result. Therefore, the approach is considered to be unsuccessful.

The results obtained by the area-based discriminant method did not express a significant difference between the second and the third groups, and the best (second) group is also inconsistent with the ground truth evaluation result either.

The evaluation results of the SSD method have achieved good efficacy in two respects. First, only the SSD method is consistent with the ground truth evaluation result. Second, among the three methods, only SSD is capable of distinguishing the significant difference of System *A* from the other. Therefore, it can be concluded that the new method can evaluate the effect of root canal preparation comprehensively and accurately. Hence, the experiment achieves the desired purpose in the dataset.

### 5.3. Robustness Evaluation

**Figure 9:**
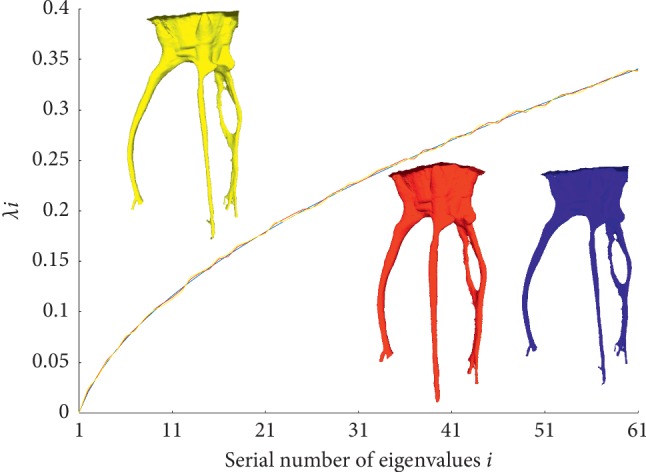
Three root canal meshes represented with 10K (blue), 50K (red), and 100K (yellow) vertices share similar Steklov spectrums.

In Figure 9, it shows an example of the robustness of our method to different sampling density. We compare the eigenvalues of a series of the root canal model with different resolution. It is observed that some geometric details in some regions are lost during the downsampling and the spectrum remains almost unaffected. According to the theory of the Steklov spectrum, the first dozens of eigenvalues and eigenfunctions are robust to downsampling for a mesh with 10k to 100k vertices. Moreover, the number of vertices is generally sufficient for shape difference evaluation.

## 6. Conclusions and Prospects

In this study, we addressed the problem of evaluating the operation result of a root canal by introducing a spectrum-based procedure called the Steklov spectrum distance, which measures the shape difference between the original shape and its ideal shape with its Steklov spectrum. We find it remarkable that the use of SSD simplifies the problem of evaluating the intrinsic and extrinsic difference between shapes, allowing to significantly improve the accuracy of standard pipelines comparing with the traditional volume-area literature. SSD appears to be a valuable tool to assess the efficacy of various root canal preparation systems.

Within the limitations of this study, one is that the error of SSD was seen in all groups, and it even diverges in the calculation of certain root canals. To solve these problems, we think that it can be optimized in two ways: one is to design a more expressive distance norm according to the specific shape of root canal; the other is to improve the numerical solution method of partial differential equation, using better presolver and matrix block parallel algorithm to improve the accuracy and efficiency of the algorithm. Another shortage to be improved is that, limited by the *μ*CT itself, this study can only be performed in vitro, rather than applied to direct clinical surgical evaluation. In the future, we will continue to consider the related research based on the data sources of high-speed acquisition of CT images in vivo.

## Figures and Tables

**Algorithm 1:**
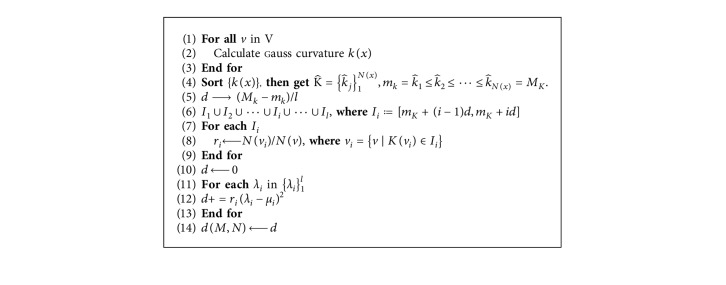
Shape evaluation based on spectral difference and Gauss curvature.

**Table 1:** Simulation environment.

Hardware types	Environment Info
CPU	Intel Core i7-3820QM CPU, 2.70 GHz
DRAM	DDR3 1600 MHz
Hard drive	2.5″ 7 mm 128 GB, 1TB, SCSI
OS	Microsoft Windows10 64 bit
IDE	Visual Studio 2012, C++
Interface design	QT4.8
Dependency library	VCGLIB/Meshlab 1.3/eigen3.0

**Table 2:** The evaluation comparison among volume method, area method, and our method for three systems (means ± SD, *n* = 20).

System			*A*	*B*	*C*	Best performance system	Consistency with ground truth evaluation result
Volume method	Mean volume of root canal (mm^3^)	Before	3.68 ± 0.92	5.02 ± 1.42	**4.18** ± **1.82**	System *C*	F
		After	4.12 ± 0.29	5.99 ± 2.01	**5.29** ± **1.12**		
		Diff.	0.44^b^	0.89^a^	**1.11** ^a*∗*^		

Area method	Mean area of root canal (mm^2^)	Before	39 ± 3.2	**36.4** ± **3.3**	33.6 ± 4.2	System *B*	F
		After	37.2 ± 4.0	**34.9** ± **3.6**	32.2 ± 3.2		
		Diff.	−1.8^a^	−**1.5** ^a^	−1.9^a*∗*^		

Our method	Mean SSD of root canal	Before	**5.9** ± **0.6**	6.5 ± 0.7	7.8 ± 1.0	System *A*	T
		After	**1.1** ± **0.2** ^a*∗*^	1.7 ± 0.3^b^	2.3 ± 0.3^b^		

Ground truth result	Mean POV	Coronal third	**2.1** ± **0.4** ^a*∗*^	3.8 ± 0.6^b^	2.9 ± 0.6^b^	System *A*	N/A
		Middle third	**1.8** ± **0.2** ^a*∗*^	2.3 ± 0.5^b^	2.1 ± 0.5^b^	System *A*	
		Apical third	**3.5** ± **0.5** ^a*∗*^	6.9 ± 0.8^b^	5.7 ± 0.9^b^	System *A*	

^*∗*^The minimum shape difference in three systems. Cells with the same letter in the superscript indicate no statistical significance between different instruments (ANOVA, Scheffé tests, *P* > 0.05).

## Data Availability

The reason why the authors choose not to disclose the data is that the research group is applying for patent at the same time, which may involve some intellectual property issues.

## References

[1] Saha P. K., Strand R., Borgefors G. (2015). Digital topology and geometry in medical imaging: a survey. *IEEE Transactions on Medical Imaging*.

[2] Orhan K., Jacobs R., Celikten B. (2018). Evaluation of threshold values for root canal filling voids in micro-CT and nano-CT images. *Scanning*.

[3] Ørstavik D. (2017). Obturation of root canals. *Endodontic Prognosis: Clinical Guide for Optimal Treatment Outcome*.

[4] Dannemann M., Kucher M., Kirsch J. (2017). An approach for a mathematical description of human root canals by means of elementary parameters. *Journal of Endodontics*.

[5] Arias A., Paqué F., Shyn S., Murphy S., Peters O. A. (2017). Effect of canal preparation with TRUShape and vortex rotary instruments on three dimensional geometry of oval root canals. *Australian Endodontic Journal*.

[6] Kierklo A., Tabor Z., Pawińska M., Jaworska M. (2015). A microcomputed tomography-based comparison of root canal filling quality following different instrumentation and obturation techniques. *Medical Principles and Practice*.

[7] Murray P. (2015). Root canal obturation. *A Concise Guide to Endodontic Procedures*.

[8] Gergi R., Osta N., Bourbouze G., Zgheib C., Arbab-Chirani R., Naaman A. (2015). Effects of three nickel titanium instrument systems on root canal geometry assessed by micro-computed tomography. *International Endodontic Journal*.

[9] Peters O. A., Paqué F. (2014). Shaping the root canal system to promote effective disinfection. *Disinfection of Root Canal Systems*.

[10] Vertucci F. J. (1984). Root canal anatomy of the human permanent teeth. *Oral Surgery, Oral Medicine, Oral Pathology*.

[11] Hammad M., Qualtrough A., Silikas N. (2009). Evaluation of root canal obturation: a three-dimensional in vitro study. *Journal of Endodontics*.

[12] Metzger Z., Zary R., Cohen R., Teperovich E., Paqué F. (2010). The quality of root canal preparation and root canal obturation in canals treated with rotary versus self-adjusting files: a three-dimensional micro-computed tomographic study. *Journal of Endodontics*.

[13] Elnaghy A. M., Elsaka S. E. (2014). Evaluation of root canal transportation, centering ratio, and remaining dentin thickness associated with ProTaper next instruments with and without glide path. *Journal of Endodontics*.

[14] Stavileci M., Hoxha V., Görduysus Ö. (2015). Evaluation of root canal preparation using rotary system and hand instruments assessed by micro-computed tomography. *Medical Science Monitor Basic Research*.

[15] Cleghorn B., Christie W., Dong C. (2007). The root and root canal morphology of the human mandibular second premolar: a literature review. *Journal of Endodontics*.

[16] Dobonagy C., Keszthelyi G., Szabo J., Sulyok P., Szabo G., Ledeczky J. (2000). A computerized method for mathematical description of three-dimensional root canal axis. *Journal of Endodontics*.

[17] Kac M. (1966). Can one hear the shape of a drum?. *The American Mathematical Monthly*.

[18] Cosmo L., Panine M., Rampini A., Ovsjanikov M., Bronstein M. M., Rodolà E. (2018). Isospectralization, or how to hear shape, style, and correspondence. https://arxiv.org/abs/1811.11465.

[19] Laga H., Guo Y., Tabia H., Fisher R. B., Bennamoun M. (2018). *3D Shape Analysis: Fundamentals, Theory, and Applications*.

[20] Hildebrandt K., Schulz C., von Tycowicz C., Polthier K. (2012). Modal shape analysis beyond Laplacian. *Computer Aided Geometric Design*.

[21] Reuter M., Spagnuolo M. Discrete Laplace-Beltrami operators for shape analysis and segmentation.

[22] Girouard A.,  Polterovich I. (2017). Upper bounds for steklov eigenvalues on surfaces. *Electronic Research Announcements in Mathematical Sciences*.

[23] Yu W., Ben-Chen M., Polterovich I., Solomon J. (2018). Steklov spectral geometry for extrinsic shape analysis. *ACM Transactions on Graphics (TOG)*.

[24] Liu J., Sun J., Turner T. (2018). Spectral indicator method for a non-self adjoint steklov eigenvalue problem. https://arxiv.org/abs/1804.02582.

[25] Girouard A., Laugesen R. S., Siudeja B. A. (2016). Steklov eigenvalues and quasiconformal maps of simply connected planar domains. *Archive for Rational Mechanics and Analysis*.

[26] Polterovich I., Sher D. A. (2015). Heat invariants of the Steklov problem. *The Journal of Geometric Analysis*.

[27] Hu J., Cao Y., Wu T., Li D., Lu H. (2014). High-resolution three-dimensional visualization of the rat spinal cord microvasculature by synchrotron radiation micro-CT. *Medical Physics*.

[28] Wang K., Huang C., Kao Y.-J., Chou C.-Y., Oraevsky A. A., Anastasio M. A. (2013). Accelerating image reconstruction in three-dimensional optoacoustic tomography on graphics processing units. *Medical Physics*.

[29] Kobayashi-Velasco S., Salineiro F. C. S., Gialain I. O., Cavalcanti M. G. P. (2017). Diagnosis of alveolar and root fractures: an in vitro study comparing CBCT imaging with periapical radiographs. *Journal of Applied Oral Science*.

[30] Berutti E., Chiandussi G., Paolino D. S. (2012). Canal shaping with waveone primary reciprocating files and ProTaper system: a comparative study. *Journal of Endodontics*.

[31] Liu J., Luo J., Dou L., Yang D. (2014). CBCT study of root and canal morphology of permanent mandibular incisors in a Chinese population. *Acta Odontologica Scandinavica*.

[32] Anbu R., Nandini S., Velmurugan N. (2010). Volumetric analysis of root fillings using spiral computed tomography: an in vitro study. *International Endodontic Journal*.

[33] Peters O. A., Laib A., Rüegsegger P., Barbakow F. (2000). Three-dimensional analysis of root canal geometry by high-resolution computed tomography. *Journal of Dental Research*.

[34] Berutti E., Paolino D. S., Chiandussi G. (2012). Root canal anatomy preservation of waveone reciprocating files with or without glide path. *Journal of Endodontics*.

[35] Ovsjanikov M., Li W., Guibas L., Mitra N. J. (2011). Exploration of continuous variability in collections of 3D shapes. *ACM Transactions on Graphics*.

[36] Fish N., Averkiou M., van Kaick O., Sorkine-Hornung O., Cohen-Or D., Mitra N. J. (2014). Meta-representation of shape families. *ACM Transactions on Graphics*.

[37] Handels H., Hacker S. (2009). A framework for representation and visualization of 3D shape variability of organs in an interactive anatomical atlas. *Methods of Information in Medicine*.

[38] Ovsjanikov M., Ben-Chen M., Solomon J., Butscher A., Guibas L. (2012). Functional maps. *ACM Transactions on Graphics*.

[39] Lipman Y., Funkhouser T. (2009). Möbius voting for surface correspondence. *ACM Transactions on Graphics (TOG)*.

[40] Rustamov R. M., Ovsjanikov M., Azencot O., Ben-Chen M., Chazal F., Guibas L. (2013). Map-based exploration of intrinsic shape differences and variability. *ACM Transactions on Graphics*.

[41] Canuto C., Hussaini M. Y., Quarteroni A., Thomas A. (2012). *Spectral Methods in Fluid Dynamics*.

[42] Mikhlin S. G. (1965). *Variational Methods in Mathematical Physics*.

[43] Auteri F., Quartapelle L. (2000). Galerkin-Legendre spectral method for the 3D Helmholtz equation. *Journal of Computational Physics*.

[44] Morse M. (1963). Harmonic extensions. *Monatshefte Fur Mathematik*.

[45] Uhlmann G. (2014). Inverse problems: seeing the unseen. *Bulletin of Mathematical Sciences*.

[46] Gilkey P. B., Grubb G. (1998). Logarithmic terms in asymptotic expansions of heat operator traces. *Communications in Partial Differential Equations*.

[47] Randolf H., Theobald F., Norman U. (2008). X-ray based methods for non-destructive testing and material characterization. *Nuclear Instruments and Methods in Physics Research Section A*.

[48] Girouard A., Polterovich I. (2014). Spectral geometry of the Steklov problem. https://arxiv.org/abs/1411.6567.

[49] Brebbia C. A., Telles J. C. F., Wrobel L. C., Mukherjee S. (1984). *Boundary Element Techniques: Theory and Applications in Engineering*.

[50] Armentano M. G., Padra C. (2008). A Posteriori error estimates for the Steklov eigenvalue problem. *Applied Numerical Mathematics*.

[51] Steinbach O. (2008). Boundary element methods. *Numerical Approximation Methods for Elliptic Boundary Value Problems*.

[52] Golub G. H., Van Loan C. F. (1996). *Matrix Computations*.

[53] Matrix S. D., Higham N. J. (1990). Analysis of the Cholesky decomposition of a semi-definite matrix. *Reliable Numerical Computation*.

[54] Huang R., Chazal F., Ovsjanikov M. (2018). On the stability of functional maps and shape difference operators. *Computer Graphics Forum*.

[55] Rustamov R. M. Laplace-Beltrami eigenfunctions for deformation invariant shape representation.

[56] Bronstein M. M. Intrinsic shape context descriptors for deformable shapes.

[57] Reuter M., Wolter F. E., Peinecke N. (2006). *Laplace-Beltrami Spectra as “Shape-DNA” of Surfaces and Solids*.

[58] Naseri M., Kangarlou A., Khavid A., Goodini M. (2013). Evaluation of the quality of four root canal obturation techniques using micro-computed tomography. *Iranian Endodontic Journal*.

